# Supplementary material for: Mindfulness and cardiovascular health: Qualitative findings on mechanisms from the mindfulness-based blood pressure reduction (MB-BP) study
Source: PLoS One. 2020 Sep 23;15(9):e0239533. doi: 10.1371/journal.pone.0239533 (PMC7510988; doi:10.1371/journal.pone.0239533)
Supplement: S1 Table — (DOCX) [file pone.0239533.s001.docx]

## S1 Table. Mindfulness-Based Blood Pressure Reduction Study: Stage 1 Qualitative Codebook

| Name | Description | Files | References |
| --- | --- | --- | --- |
|  |  |  |  |
| **Mindfulness Practice** | *This category refers to the range of specific mindfulness practices (breath, yoga, etc.)* | 10 | 93 |
| Body Awareness | *Body awareness or interoceptive awareness refers to the practice of becoming aware of physical sensations (e.g. tingling, numbness, heartbeat) within the body as a focus for awareness training. Informal practices of the “body scan” were also coded as body awareness. For example, participants who indicated that they engaged in impromptu awareness or scanning of physical sensations were defined as engaging in “body awareness” however this would also be considered an informal “body scan” practice.* | 6 | 14 |
| Body Scan | *Refers to the systematic targeting of awareness from one are of the body to another (e.g. foot to ankle, ankle to thigh). Body scanning was defined as formal and systematic however, informal scanning can occur and was identified as “body awareness.”* | 3 | 7 |
| Breath Awareness | *Practices in which participants focus directly on the breath (e.g. sensations of air flowing in, down through the nose out through the mouth).* | 6 | 16 |
| External Awareness | *Practices relating to directing and maintaining attention on an external stimulus (e.g. sound, visuals).* | 2 | 2 |
| Gratitude Practice | *Practices involving directing kindness/friendship and well-being hope for themselves and others.* | 2 | 5 |
| Lovingkindness | *This can be the actual act of eating (chewing swallowing) or mindfulness of what the participant was putting in their body.* | 4 | 6 |
| Mindfulness of Eating | *Instances of attention and awareness were participants became aware of thoughts, emotions, physical sensations, and external events in the present moment (e.g. the physical manifestation of stress).* | 5 | 8 |
| Walking Meditation | *Practices in which the attentional focus is on the sensations and experiences associated with walking (e.g. lifting the foot, shifting the knee, setting the foot down).* | 4 | 6 |
| Yoga Practice | *Practices relating to yoga either those that were taught in class or practices the participant engaged in outside of the course.* | 5 | 11 |
| **Self-Regulation Framework** | *Attention control Self-awareness Emotional Regulation are all defined as components that enhance and contribute to increase self-regulation.* | 7 | 60 |
| Attention Control | *The practice and cultivation of attention. Attention is often subdivided into three components: alerting (readiness in preparation for an impending stimulus, which includes tonic effects that result from spending time on a task (vigilance) and phasic effects that are due to brain changes induced by warning signals or targets); orienting (the selection of specific information from multiple sensory stimuli); and conflict monitoring (monitoring and resolution of conflict between computations).** | 3 | 4 |
| Emotional regulation | *Emotion regulation refers to strategies that can influence which emotions arise and when, how long they occur, and how these emotions are experienced and expressed. A range of implicit and explicit emotion regulation processes has been proposed, and mindfulness- based emotion regulation may involve a mix of these processes, including attentional deployment, cognitive change and response.** | 5 | 18 |
| Self-Compassion | *Self-compassion refers to the ability to non-judgmentally stay with unpleasant thoughts, physical sensations, or emotional states understanding that these are impersonal states that all people experience while wishing to alleviate this suffering through positive action.* | 1 | 1 |
| Self-Kindness | *Acts of kindness and care directed from an individual toward themselves. May be thoughts, emotional considerations, or actual behavioral response. May be originate as a way to alleviate some painful or difficult event but these are not necessary for the presentation of a kind act.* | 2 | 5 |
| Self-Awareness | *Awareness of present moment experiences and additional self-referential processing including positive reappraisal of self, awareness of breath, heartbeat, and other physical sensations or emotional/cognitive events.** | 4 | 33 |
| Self-Care practice | *The concept of engaging in an activity for an individual’s health and well-being may be motivated by desire for good health or to show kindness to oneself.* | 3 | 5 |
| **Stress Management** | *Instances when tools learned in the program are used for stress management.* | 8 | 30 |

** Tang YY, Holzel BK, Posner MI. The neuroscience of mindfulness meditation. Nat Rev Neurosci. 2015;16(4):213-225.*
