# Supplementary material for: Mindfulness and cardiovascular health: Qualitative findings on mechanisms from the mindfulness-based blood pressure reduction (MB-BP) study
Source: PLoS One. 2020 Sep 23;15(9):e0239533. doi: 10.1371/journal.pone.0239533 (PMC7510988; doi:10.1371/journal.pone.0239533)
Supplement: S1 File — (DOCX) [file pone.0239533.s002.docx]

## S1 File. Focus Group Protocol

| Agenda | Length of Section |
| --- | --- |
| Research assistant downstairs directing participants. Set up name tents.  Test recorder.  Turn on recorder, place in the middle of the table. | **Start** |
| Chatting and eating. | **15 minutes** |
| Welcome and introduction   - Thank you for sharing your thoughts on MB-ICR - Introductions   - Qualitative Research Specialist   - Research Assistant - Why and How   - We are trying to improve the intervention   - Discussing your opinions on the different activities   - Please share your point of view, even if it’s different from others   - No wrong answers   - We equally welcome positive and negative feedback - Suggestions   - Suggestions to help us have a good discussion     - Speak up     - Audio recording, one person speaks at a time     - Any report that we write about what we hear today will not be associated with your identity     - Tendency for some people to be comfortable speaking up more than others. It’s important to us to hear from everyone today. So, I may ask you to share if I haven’t hear from you. Or I may ask you to let others share if you are sharing a lot. - What to expect   - My role is to listen, facilitate discussion between you all and move us along 5 questions.   - In the interest of time, I may have to move the discussion along so that we can cover all 5 questions   - Let’s begin | **20 minutes** |
| Allow participants to look over class overview.  Looking at the list of course activities, which is most memorable for you? Why? | **5 minutes** |
| Give them cards showing different activities.  Put cards for activities you found very useful in one pile, modules that you thought were somewhat useful in another pile, and activities you thought were not useful in a 3rd pile.  Please place the cards in the piles according to whether you found them “very useful,” “somewhat useful,” or “not useful.” | **10 minutes**  **5 minutes** |
| Research assistant writes the questions shown below on the board.  1. What was most helpful about this course, and why? | **15 minutes** |
| 2. After going through this mindfulness intervention, what is your understanding of how it works to improve your cardiovascular health? | **20 minutes** |
| 3. We want to make this intervention better. You have been through it once. How do you think we can make it better? | **10 minutes** |
| 4. Every instructor can improve. How can this instructor improve? | **10 minutes** |
