# Supplementary material for: Mindfulness and cardiovascular health: Qualitative findings on mechanisms from the mindfulness-based blood pressure reduction (MB-BP) study
Source: PLoS One. 2020 Sep 23;15(9):e0239533. doi: 10.1371/journal.pone.0239533 (PMC7510988; doi:10.1371/journal.pone.0239533)
Supplement: S2 File — (DOCX) [file pone.0239533.s003.docx]

## S2 File: In-Depth Interview Protocol

| Agenda | Length of Section |
| --- | --- |
| Ensure zoom call is set-up correctly  Confirm that all documentation required is available (e.g. informed consent, descriptions of intervention activities, | **Pre-call Preparation** |
| Phone greeting: Hello my name is ___________ I am calling from the Mindfulness-Based Blood Pressure Reduction Study. I’m calling to conduct the interview ___________ had previously scheduled with you. | **2 minutes** |
| - Informed consent: participant is read the informed consent document and asked to provide verbal confirmation consistent with IRB approved protocol. - Thank you for sharing your thoughts on MB-BP - Why and How   - We are trying to improve the intervention   - Discussing your opinions on the different activities   - Please share your point of view, even if it’s different from others   - No wrong answers   - We equally welcome positive and negative feedback - Suggestions   - Suggestions to help us have a good discussion     - Speak up     - Zoom recording using audio.     - Any report that we write about what we hear today will not be associated with your identity     - Tendency for some people to be comfortable speaking up more than others. It’s important to us to hear from everyone today. So, I may ask you to share if I haven’t hear from you. Or I may ask you to let others share if you are sharing a lot. - What to expect   - My role is to listen, facilitate discussion between you all and move us along 5 questions.   - In the interest of time, I may have to move the discussion along so that we can cover all 5 questions   - Let’s begin | **5 minutes** |
| Read participant class overview remind them this has been emailed in case they would like to review again.  Looking at the list of course activities, which is most memorable for you? Why? | **5 minutes** |
| Next we have a series of four questions about the program we’d like to as you. The first is…  1. What was most helpful about this course, and why? | **5 minutes** |
| 2. After going through this mindfulness intervention, what is your understanding of how it works to improve your cardiovascular health? | **5 minutes** |
| 3. We want to make this intervention better. You have been through it once. How do you think we can make it better? | **5 minutes** |
| 4. Every instructor can improve. How can this instructor improve? | **5 minutes** |
| Is there any additional feedback you would like to provide? | **3-5 minutes** |
| Thank them for participation and confirm method of reimbursement Email/Mail | **2 minutes** |
